# Helsinki VideoMEG Project: Augmenting magnetoencephalography with synchronized video recordings

**DOI:** 10.1016/j.mex.2018.01.002

**Published:** 2018-01-31

**Authors:** Andrey Zhdanov, Jussi Nurminen, Eric Larson

**Affiliations:** a BioMag Laboratory, HUS Medical Imaging Center, Hospital District of Helsinki and Uusimaa, P.O. Box 340, FI-00029, Finland; b University of Washington, Institute of Learning and Brain Sciences, 1715 NE Columbia Road, Box 357988, Seattle, WA 98195, USA

**Keywords:** Video-magnetoencephalography (Video-MEG), Biomagnetics, Magnetoencephalography, Epilepsy, Video recording

## Abstract

The primary goal of the Helsinki VideoMEG Project is to enable magnetoencephalography (MEG) practitioners to record and analyze the video of the subject during an MEG experiment jointly with the MEG data. The project provides:

• Hardware assembly instructions and software for setting up video and audio recordings of the participant synchronized to MEG data acquisition.
• Basic software tools for analyzing video and audio together with the MEG data.

The resulting setup allows reliable recording of video and audio from the subject in various real-world usage scenarios. The Helsinki VideoMEG Project allowed successful establishment of video-MEG facilities in four different MEG laboratories in Finland, Sweden and the United States.

## Graphical abstract

**Figure:**
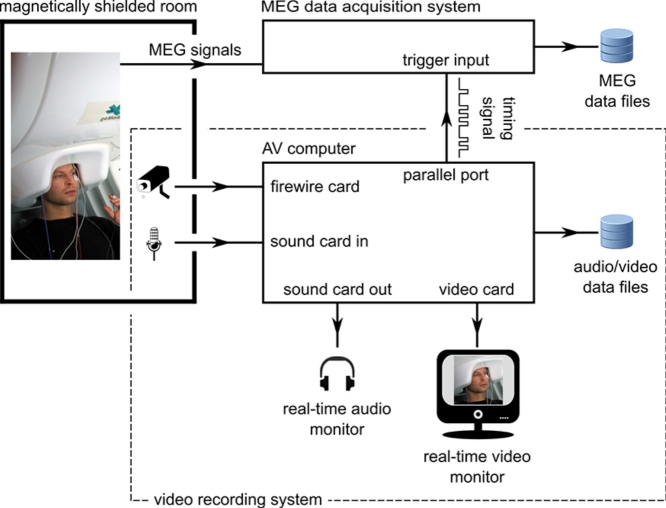

## Method details

Magnetoencephalography (MEG) is a non-invasive functional brain imaging method that monitors neuronal activity by measuring the associated magnetic fields [1]. In clinical practice MEG is mostly used for pre-surgical localization of epileptogenic zones, where it has been shown to detect sources of pathological activity that are undetectable by other non-invasive techniques [[2], [3]].

MEG is in many respects similar to the closely related technique of electroencephalography (EEG): the two techniques share the underlying sources of the signal, and rely on similar mappings from the activity of these sources to the signals measured by the sensors, yielding similar temporal and spatial resolutions [1]. In clinical practice, EEG examinations of epilepsy patients are routinely augmented with synchronized video recordings [4]—a procedure known as video-EEG or VEEG. In video-MEG—the MEG counterpart of the video-EEG procedure—of epilepsy patients, video has proven useful for identifying artifacts and documenting seizures [5]. Adding time-synchronized video to MEG epilepsy recordings has been demonstrated to significantly affect the interpretation of the data [6]. In addition to clinical applications, video recording of the subject can also be useful in basic research, for example, for verification of subject compliance and performance.

Despite the potential benefits, video-MEG recordings have gained little traction with MEG practitioners. One of the main impediments to wider adoption of video-MEG is the lack of practical solutions that would allow an MEG laboratory to integrate video recordings into the workflow with reasonable cost and manpower requirements. While several MEG manufacturers have advertised integrated video recording capabilities in their future products, currently there are no commercial video-MEG solutions on the market. Moreover, these advertised capabilities are only available for new MEG device installations, which makes them irrelevant to existing MEG sites.

## Project's goals and philosophy

The Helsinki VideoMEG Project described in this paper aims at remedying this situation by providing MEG practitioners with tools for setting up video-MEG recordings. The project is guided by three main principles:

1. **Practicality.** The project's goal is to allow a typical MEG facility to establish a video-MEG operation *in practice* with reasonable monetary and manpower costs.
2. **Openness.** All the materials (software, documentation, installation instructions, etc.) are freely available on GitHub^1^ under an open-source license. The users are free to study, redistribute, and modify these according to their needs. The users are also welcome to contribute modified versions of the materials back to the project.
3. **Vendor- and device-neutrality.** The project aims at providing a video-MEG solution that is compatible with any MEG device.

Establishing a video-MEG operation entails two relatively separate developments: (1) creating an instrumentation setup for recording video and audio of the participant during the MEG experiment in a way that is synchronized to MEG data acquisition, and (2) setting up a facility for the analysis of the resulting video, audio, and MEG data streams.

The first task—establishing a video-MEG recording setup—necessarily involves acquisition and installation of video recording hardware. To put video-MEG within a reach of a typical MEG facility, the Helsinki VideoMEG Project has developed a hardware setup that only uses widely available standardized off-the-shelf components and requires no special skills for assembling. The project provides all the material (assembly and installation instructions, software, etc.) necessary for setting up video-MEG recordings.

The second task, setting up a video-MEG analysis facility, presents a different set of challenges. On one hand, it does not require any hardware development, as it can be completely addressed by developing appropriate software tools. On the other hand, integration with the existing instruments poses much bigger challenge for video-MEG analysis than it does for video-MEG recordings. Currently, MEG practitioners use a whole spectrum of different tools for analyzing the data. These include open-source packages, such as FieldTrip [7] or MNE [8], that provide a lot of power and flexibility at the expense of ease-of-use, as well as closed proprietary Graphical User Interface (GUI)-based software suits heavily optimized for relatively narrow range of workflows, such as Elekta Oy's DANA software. The latter class of MEG analysis tools is especially popular with clinicians, who constitute an important target audience for video-MEG. Ideally, the Helsinki VideoMEG Project should provide software that integrates video analysis capabilities into existing MEG analysis tools, however, in practice this is next to impossible for proprietary software. The project therefore adopts a pragmatic approach of adding video functionality to existing software tools when it is possible, while relying on workarounds when it is not. For the MEG users that use MATLAB- or Python-based scientific computing environments for their data analysis, the project offers MATLAB and Python routines for importing video and audio data and synchronizing it to MEG traces. For MEG practitioners that rely on proprietary GUI-based tools for interactive exploration of the data, the project provides several basic standalone utilities that can be used in conjunction with the user's favorite MEG analysis software.

## Video-MEG recording

For recording video and audio of the participant, the Helsinki VideoMEG Project employs video recording system, which essentially constitutes a separate instrument that is independent of the MEG device. The only link between the two is the synchronization line that carries the timing information encoded as a sequence of trigger pulses. Thus, any MEG device capable of recording external trigger pulses can be used with the system.

**Fig. 1:**
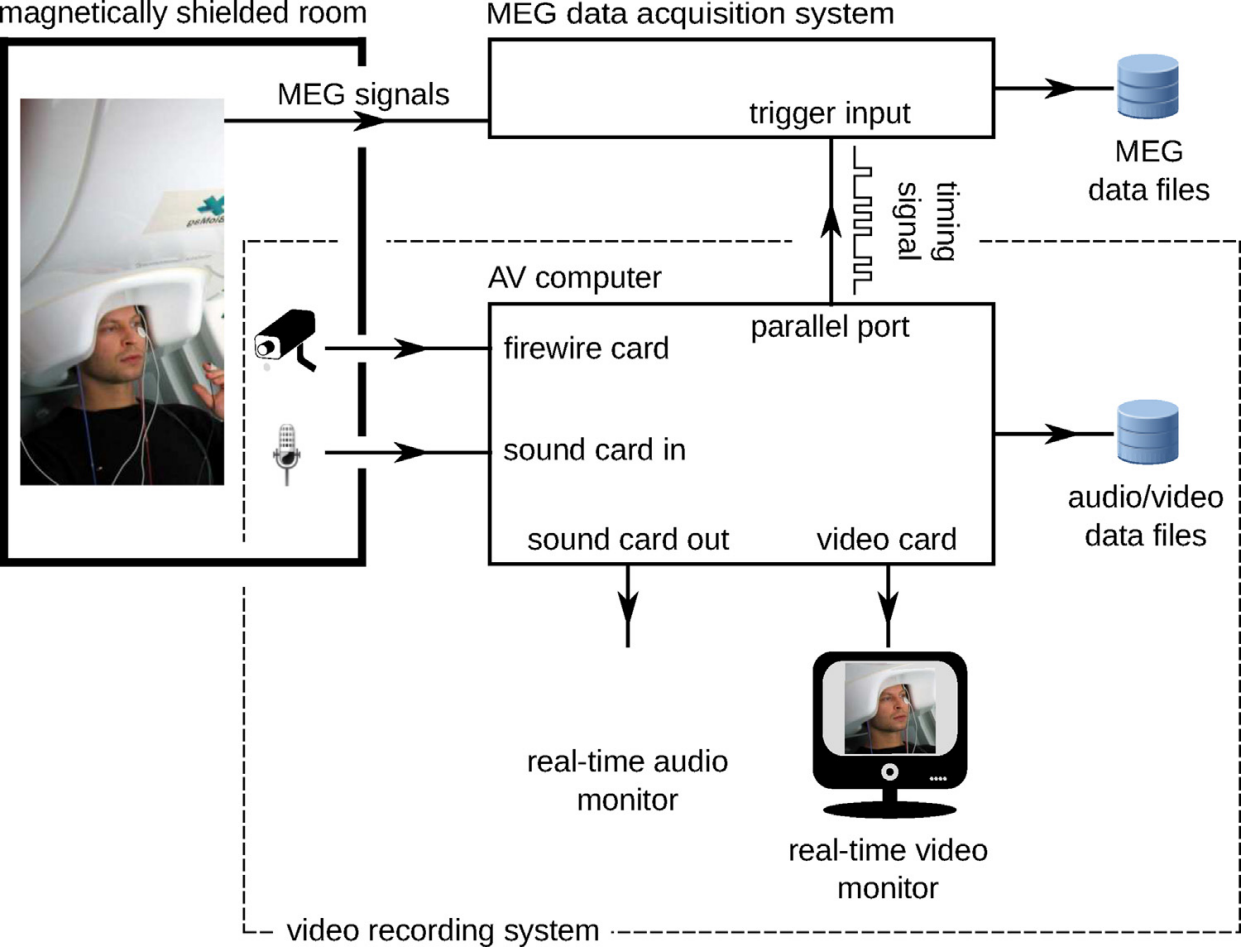
Overview of the video recording system.

Fig. 1 provides an overview of the video recording setup. Audio and video of the patient are captured with a microphone and one or more video cameras located inside the magnetically shielded room (MSR). The microphone and the cameras are connected to the audiovisual (AV) computer. The AV computer timestamps the audio and video data and stores it as files on the computer's local hard drive. In addition, the AV computer generates timing trigger sequences that are recorded by the MEG device and used for synchronizing MEG traces to the video and audio streams.

### AV computer and software

The AV computer is a standard amd64-based office PC running a 64-bit version of Linux operating system. The setup was tested with Ubuntu 14.04 LTS and Ubuntu 16.04 LTS distributions of Linux, but other distributions may work as well. The computer needs to be equipped with a parallel port interface for outputting timestamps and an IEEE 1394 (firewire) interface for connecting the cameras. The firewire link can use either a standard copper cable or an optical fiber for communicating with the cameras, requiring either a standard or an optical interface, respectively. The AV computer runs custom Qt-based software written in C++. The software monitors and records the video and audio streams, and generates the timing signals that are emitted over the computer's parallel port.

### Cameras and microphone

The project supports firewire machine-vision cameras that implement the industry-standard protocol—IIDC 1394-Based Digital Camera Specifications, also known as the DCAM^2^—for communicating with the host computer. Either grayscale or color cameras can be used. By default, the software records in a resolution of 640 × 480. If the cameras use an optical fiber for the link to the AV computer, they need a separate power supply, which would typically be located inside the RF-shielded stimulation cabinet of the MEG system. Cameras can be daisy-chained. Currently, the software supports up to six cameras; the performance of the AV computer may also limit the usable number of cameras, depending on the chosen resolution and frame rate. Especially in epilepsy recordings, several cameras can be useful as they provide a better overview of the patient [5].

The AV computer allows recording the audio from inside the MSR via the computer's sound card. The exact configuration of the audio recording setup depends on the site's specific requirements. One possible choice (easy to install but quite expensive) is an optical microphone, such as Sennheiser MO 2000 (Sennheiser, Hanover, Germany). The microphone itself does not contain any magnetic parts, and the signal connection is optical, eliminating any sources of interference inside the MSR. For a low-cost alternative, an electret microphone can be used.

### Timing and synchronization

**Fig. 2:**
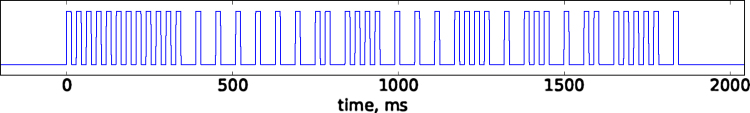
One timestamp as recorded on the trigger channel of the MEG device. Individual bits of the timestamp are encoded as delays between subsequent pulses.

The system uses the Unix time (POSIX time) in milliseconds as timestamps. The recording software timestamps every audio buffer and video frame as soon as they are available. The timestamps are also sent to the parallel port at regular intervals (by default every 10 s). They are represented as sequences of constant-amplitude pulses, where bits are encoded as delays between subsequent pulses’ rising edges (Fig. 2). Values 0 and 1 are represented as short and long delays (by default 30 and 60 ms) respectively. Altogether 42 bits are used to represent the POSIX timestamp in milliseconds. The sequence starts with the least significant bit and ends with a parity bit, with a total of 43 bits.

Typically the parallel port output is connected to an MEG trigger channel, which is recorded synchronously with other MEG data. Thus a timestamped MEG sample is available every 10 s. In postprocessing, the timestamps for MEG samples between the timing pulses can then be computed by linear interpolation.

### Audio and video file formats

The video is recorded as individual JPEG-encoded frames; no intraframe coding is currently performed. Thus, individual frames can be easily retrieved independently of each other at the expense of increased disk usage. The JPEG frames are stored consecutively in a single file per camera, interleaved with the timestamps for each frame.

The audio is recorded as buffers. Buffer size may depend on the particular sound card and drivers used; a typical buffer size is 1024 samples, corresponding to about 23 ms at a typical sampling rate of 44,100 Hz. Similar to video, audio buffers are stored into a single file, interleaved with their corresponding timestamps.

The exact specification of the audio and video file formats is available from the project's website.

## Video-MEG analysis

The Helsinki VideoMEG Project approaches the task of analyzing video-MEG recordings in two ways described below.

### MATLAB and Python routines

For the users implementing their own analysis pipeline in MATLAB or Python programming environments, the project offers a complete set of functions for integrating audio and video streams into the analysis, namely routines for loading the audio and video data, and extracting and interpolating timestamps.

Additionally it provides utility programs, written in Python, for various ancillary tasks—e.g. exporting video and audio to standard AVI format. The project also offers several documented examples of simple analysis pipelines written in Python.

### GUI-based analysis software

At the current stage, the selection of GUI-based tools for interactive review of the video-MEG data is quite limited. Elekta Oy has demonstrated an experimental version of it's Graph software that allows review of video and audio jointly with the MEG data from the company's VectorView and Triux MEG devices [6]. This software, however, has never been officially released by Elekta and it's availability to a typical MEG facility is uncertain.

**Fig. 3:**
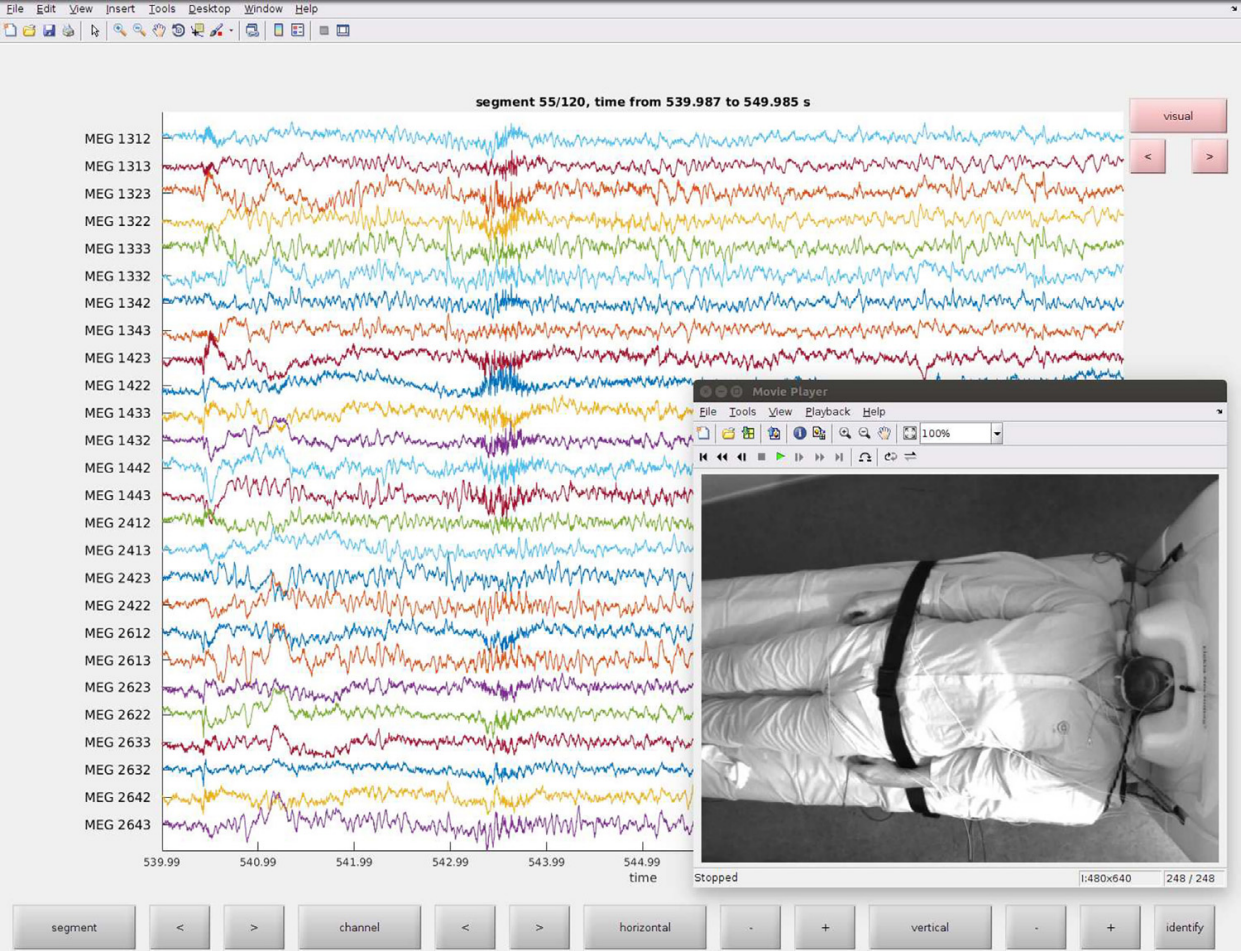
FieldTrip provides a basic tool for interactive inspection of video and MEG/EEG data.

The developers of the FieldTrip software [7] have added initial support of the Helsinki VideoMEG Project's video and audio formats to their package. This provides FieldTrip users with a basic tool for interactive review of MEG and EEG traces jointly with video and audio (see Fig. 3).

Notwithstanding the aforementioned options, the lack of a practical GUI-based video-MEG data analysis tool currently constitutes the main hindrance to the project's progress and the main focus of the future development roadmap.

## System performance—synchronization accuracy

We performed a test measurement to assess the accuracy of synchronization between the audio and video streams and the MEG data. The test system consisted of a Dell Precision 490 office PC (Dell Corporation, Round Rock, Texas, USA) running the Ubuntu Linux 14.04 operating system. The motherboard integrated sound system based on the Sigmatel STAC9200 chip was used for audio. For video, two Allied Vision Stingray F033 cameras (Allied Vision Technologies GmbH, Stadtroda, Germany) were connected to the system via optical firewire in a daisy-chained configuration.

The Elekta TRIUX system (Elekta Oy, Helsinki, Finland) in the BioMag laboratory of Helsinki University Central Hospital was used as the MEG device. The MEG was set up to generate trigger pulses once per second. The video cameras were pointed at the trigger interface unit which has LEDs indicating trigger onset. A function generator was set up to output a 1-kHz sinusoidal pulse with a 100-ms duration on the rising edge of the trigger signal. Using an oscilloscope, we verified that the delay between the trigger rising edge and the sinusoid onset was below 1 ms. The output of the function generator was then connected to the line input of the soundcard. Thus the rising edge of the pulse served as a reference event used for testing the synchronization between the three data streams—MEG, video and audio. Each rising edge (which was recorded in the MEG data as a trigger) coincided with the onset of the LED flash (which was recorded on video) and onset of the sinusoidal pulse (which was record on audio). Data were acquired continuously for 20 min.

Next, the data were synchronized using the VideoMEG Python utilities. After synchronization, tone onsets were localized from the recorded audio data by a correlation with a 1-kHz complex exponential and thresholding. LED onsets were detected from the video data by thresholding: the frame where the intensity of the LED first reached 50% of its maximum was denoted as the LED onset frame. Since the LED may be on for only a part of the video camera's frame acquisition interval, the on/off transition is not necessarily instantaneous in the video.

If the synchronization between video, audio, and MEG traces were perfect, the reference event—rising edge of the trigger pulse—would be assigned the same time in all the three data streams. The extent of the discrepancies between the timings assigned to the same event in different data streams characterize the accuracy of synchronization.

**Fig. 4:**
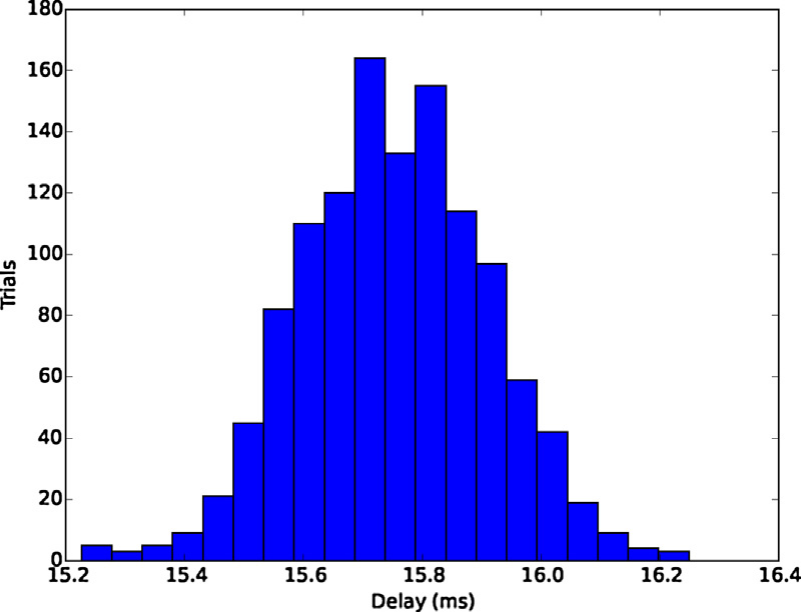
Trigger-to-onset delay for audio in the test recording.

Fig. 4 shows the histogram of the discrepancies between audio and MEG timings of the reference events. The mean discrepancy was 15.8 ms with a standard deviation 0.157 ms. Discrepancies between video and MEG timings of the events varied between 0 and 1 frames. In other words, following a trigger event, the LED was detectable either immediately in the next available frame or the one after that. The probabilities for 0-frame delay were 11.6% and 12.0% for cameras 1 and 2, respectively. Thus most of the LED flashes were detected with 1-frame delay (88.4% and 88.0% for cameras 1 and 2, respectively).

## Discussion

We have presented the architecture of a low-cost video recording system whose output can be accurately synchronized with the rest of the MEG data stream. The total cost of the system hardware depends on the number and type of cameras and on whether an optical microphone is used; a basic system can be put together for approximately 5000 €.

On our setup based on a standard office PC, the system was verified to have relatively short audio and video latencies. Critically, audio and video jitter were small: <1 ms for audio and 1 frame for video in this hardware setup. The fixed audio delay of approximately 16 ms should be low enough for clinical applications. However, for applications demanding maximally accurate synchronization of the external audio stream (e.g. neuroscience experiments), we recommend compensating for the fixed delay. This is possible as long as the jitter is negligible. The delay and jitter might depend on the particular hardware used, so we recommend validating individual setups before deployment.

Below we outline the rationale for some of the project's design decisions.

### Video-MEG synchronization mechanism

To synchronize the AV computer to the MEG, the project relies on binary timing signals from the AV computer recorded by the MEG devices’ trigger channel. This approach offers the advantages of being simple and reliable, and, importantly, independent of the internals of the MEG acquisition system. The only requirement for the MEG device is to be able to accurately register external trigger pulses, which in practice is satisfied by any modern MEG system.

Encoding the absolute time in a sequence of pulses every 10 s (rather than, for example, producing a single pulse every 10 s) significantly increases the robustness of the synchronization scheme. It allows synchronization in the presence of partially missing or corrupt data and prevents accidental missynchronization of unrelated data streams. The redundancy of such an encoding, complemented by the redundancy provided by parity bits, allows reliable automatic detection of the timing information in the MEG data without the need for the user to explicitly specify the timing channel.

To emit the timing signal, the AV computer requires a binary output facility that can produce an arbitrary sequence of pulses and allow accurate enough control of their timing. Whereas there is a multitude of options available for solving this problem—such as digital-to-analog converters, digital input/output cards, etc.—by far the cheapest, simplest and easiest solution employs parallel (also known as LPT) port. Although the LPT technology is obsolete, parallel ports are still widely available for desktop computers as add-on PCI cards.

### The choice of video cameras

When selecting a type of camera to be used for recording the patient, we have considered a number of possible alternatives, such as consumer web-cameras, surveillance cameras, and industrial machine-vision cameras. We have finally decided to adopt firewire-based machine vision camera a number of reasons:

• Such cameras offer much longer product life cycle than most of the alternatives. That means that the cameras, associated materials (drivers, documentation, power supplies, etc.), and technical support will be available for many years into the future.
• The cameras rely on open and stable IIDC interface for communicating with the host computer. This allows to avoid a vendor/model lock-in, as the camera can be easily replaced with another one from a wide selection of models by different vendors that support the interface.
• Being designed for integration into third-party systems, machine-vision cameras offer the user an extensive control over the camera configuration both physical (such as lens type, presence or absence infrared cut filter, mounting attachments) and programmatic (for example, the software control of all the video acquisition parameters—exposure time, frame rate, etc.). This level of control is critical to the project's ability to accommodate a wide range of requirements posed by different clinical and research use cases—such as the demand for recordings in low levels of visible light or different experiment-specific requirements for video quality and frame rate.
• Some firewire-based machine vision cameras (for example, those in Stingray and Pike model lines by Allied Vision Technologies) allow the use of optical fiber for data connection to the host computer instead of the standard copper firewire cable. The cameras still require a conductive cable for supplying the power, however the power supply can be completely independent from the host computer. This greatly simplifies the practicalities of installing the camera inside the MSR. Using an optical fiber instead of a copper cable may also reduce the amount of electromagnetic interference introduced by the setup, however, this was not systematically tested.

For the initial clinical validation we constructed a prototype that employs two black-and-white Stingray F-033 cameras at resolution 640 × 480 and frame rates of either 30 or 60 fps. This choice of a relatively low-end camera model was motivated by the intent to minimize the costs and avoid potential performance problems. In line with previous reports [[5], [9]], our experience confirms that even at this modest resolution video provides significant additional clinical value in MEG examinations of epilepsy patients [6]. However, the Helsinki VideoMEG Project can accommodate cameras with higher resolutions, albeit at the expense of higher requirements for the AV computer hardware and storage space. With firewire cameras, for a given frame rate the video resolution is ultimately limited by the firewire bandwidth; however, this limit is way above our current resolution of 640 × 480—there are cameras that attain the resolution of 1388 × 1038 at 30 fps for color video (Pike F-145 by Allied Vision Technologies) and full HD (1920 × 1080) at 30 fps for black-and-white video (Pike F-210B by the same manufacturer).

### Video and audio data formats

The project employs a simple custom format for storing video and audio data. The audio is stored uncompressed and video—as a sequence of JPEG-compressed individual frames. In the initial stages of the project, we prefer a custom format over commonly used ones (such as, for example, MPEG) because it considerably simplifies the development of video recording and analysis tools. The main drawbacks of using a simple custom data format are: (1)unavailability of the data for analysis by numerous third-party tools, such as video editors, and (2) the increase in storage requirements (due to a very simple compression scheme).

The address the first issue, the project provides conversion utilities that allow export of the video and audio data to a widely-used AVI format.

For the default camera resolution currently provided by the project, the second shortcoming is tolerable, if not negligible. The amount of storage space required by video and audio is comparable to that required by MEG data, thus most MEG sites should be able to handle the additional storage demands effected by the video without any significant modifications to their data storage facilities. This situation is, however, bound to change as the project embraces higher-resolution cameras. Hence, transitioning to a more sophisticated video storage format is one of the projects near-term goals.

## Conclusion

With the exception of a comprehensive GUI-based analysis tool, the Helsinki VideoMEG Project provides a complete set of instruments for integration of video and audio recordings of the subject into the MEG laboratory workflow.

## Footnotes

2 See http://1394ta.org for details.

## Footnotes

1 https://github.com/andreyzhd/VideoMEG.
